# Comparison of Multi-Frequency and Multi-Coil Electromagnetic Induction (EMI) for Mapping Properties in Shallow Podsolic Soils

**DOI:** 10.3390/s20082330

**Published:** 2020-04-19

**Authors:** Daniel Altdorff, Kamaleswaran Sadatcharam, Adrian Unc, Manokarajah Krishnapillai, Lakshman Galagedara

**Affiliations:** School of Science and the Environment, Memorial University of Newfoundland, Corner Brook, NL A2H 5G4, Canada; ksadatcharam@grenfell.mun.ca (K.S.); aunc@grenfell.mun.ca (A.U.); mkrishna@grenfell.mun.ca (M.K.); lgalagedara@grenfell.mun.ca (L.G.)

**Keywords:** multi-coil EMI, multi-frequency EMI, comparative study, proximal soil sensing, noninvasive mapping, instrument selection, podzol

## Abstract

Electromagnetic induction (EMI) technique is an established method to measure the apparent electrical conductivity (EC_a_) of soil as a proxy for its physicochemical properties. Multi-frequency (MF) and multi-coil (MC) are the two types of commercially available EMI sensors. Although the working principles are similar, their theoretical and effective depth of investigation and their resolution capacity can vary. Given the recent emphasis on non-invasive mapping of soil properties, the selection of the most appropriate instrument is critical to support robust relationships between EC_a_ and the targeted properties. In this study, we compared the performance of MC and MF sensors by their ability to define relationships between EC_a_ (i.e., MF–EC_a_ and MC–EC_a_) and shallow soil properties. Field experiments were conducted under wet and dry conditions on a silage-corn field in western Newfoundland, Canada. Relationships between temporally stable properties, such as texture and bulk density, and temporally variable properties, such as soil water content (SWC), cation exchange capacity (CEC) and pore water electrical conductivity (EC_w_) were investigated. Results revealed significant (*p* < 0.05) positive correlations of EC_a_ to silt content, SWC and CEC for both sensors under dry conditions, higher correlated for MC–EC_a_. Under wet conditions, correlation of MF–EC_a_ to temporally variable properties decreased, particularly to SWC, while the correlations to sand and silt increased. We concluded that the MF sensor is more sensitive to changes in SWC which influenced its ability to map temporally variable properties. The performance of the MC sensor was less affected by variable weather conditions, providing overall stronger correlations to both, temporally stable or variable soil properties for the tested Podzol and hence the more suitable sensor toward various precision agricultural practices.

## 1. Introduction

Characterization of spatiotemporal variability of relevant physicochemical properties of soil is crucial for precision agriculture and for various environmental sectors [1]. Commonly, soil sampling and laboratory analyses are carried out to understand the spatiotemporal variability of soil properties. However, conventional methods involve invasive soil sampling which is expensive and time consuming and only provide point information. Moreover, soil sampling is often technically not feasible for large-scale and extended temporal monitoring or for areas with restricted accessibility [2,3,4,5]. Mapping of proxy properties, such as apparent electrical conductivity (EC_a_) by electromagnetic induction (EMI) allows for an indirect, cost effective, and non-invasive mapping of relevant soil properties over larger areas (e.g., 30 ha [6,7]). In addition, non-invasive in situ techniques may allow a reduction in the excessive use of environmentally unfriendly chemical-based laboratory analyses.

EC_a_ as recorded by EMI has been used as a proxy for various soil properties for several decades. The relationship of EC_a_ to relevant soil properties, such as soil texture [8], clay content [9], soil water content—SWC [10], soil organic matter—SOM [11], bulk density—BD [12,13], cation exchange capacity—CEC [14,15], and pore water electrical conductivity—EC_w_ [16] are well documented. However, the correlations of EC_a_ to relevant soil properties are still complex and test-site dependent given that EC_a_ values are integrative parameters, affected by soil depth heterogeneity, which hinders the allocation of EC_a_ values to a certain soil property. Although the depth of signal origin from multi-frequency (MF) and multi-coil (MC) EMI sensors and the effecting properties are theoretically defined, they can highly vary under heterogeneous field conditions. Hence, understanding the differences between the EC_a_ as recorded by MF and MC sensors can help to select the best instrument for non-invasive mapping of the targeted soil properties. Overall, EC_a_ variations measured under non-saline soil conditions are primarily associated with the variables such as texture (particularly clay and silt content), SWC, porosity, EC_w_, and CEC [3,17,18,19]. From the affecting variables, SWC has been shown to be the dominant parameter governing variability in EC_a_ in soils with very low clay content, and resulting low CEC, such as Orthic Humo-Ferric Podzol found in western Newfoundland [2]. 

Given the prominent role of SWC, a comparative study for the assessment of MF and MC sensors should be executed under both, dry and wet weather conditions. Moreover, as knowledge on the spatio-temporal variability of SWC is of particular interest for agricultural and environmental processes [20], the comparative investigation should highlight the capability of each sensor to map SWC variations. Although podzol is commonly regarded as not suitable for agricultural use [21], it is expected to become much more agronomically suitable, as climatic zones and agricultural production is shifting northwards [22,23,24]. Hence, understanding of processes and properties associated with podzolic soils are of particular importance for their projected future use for agriculture. Here, we investigated the performance of MF and MC sensors for their ability to map shallow soil properties as relevant for agricultural management in a boreal podzolic soil. Based on the claims of the manufacturers and previous studies, we hypothesized that both instruments are suitable to collect EC_a_ measurements equally for establishing correlations to temporally stable and variable soil properties. Given the prominent role of SWC among the influencing variables as highlighted, we also hypothesized that the correlation of EC_a_ to other variables will be lower under wet conditions because of the deflecting influence of SWC on all correlations. Likewise, we expected that higher SWC will increase its correlations to all EC_a_ readings from both sensors. We further hypothesized that the correlations as obtained by the two sensors will be similar and sufficiently accurate to predict the spatial variation of the targeted soil properties.

## 2. Materials and Methods

### 2.1. Study Area

**Figure 1:**
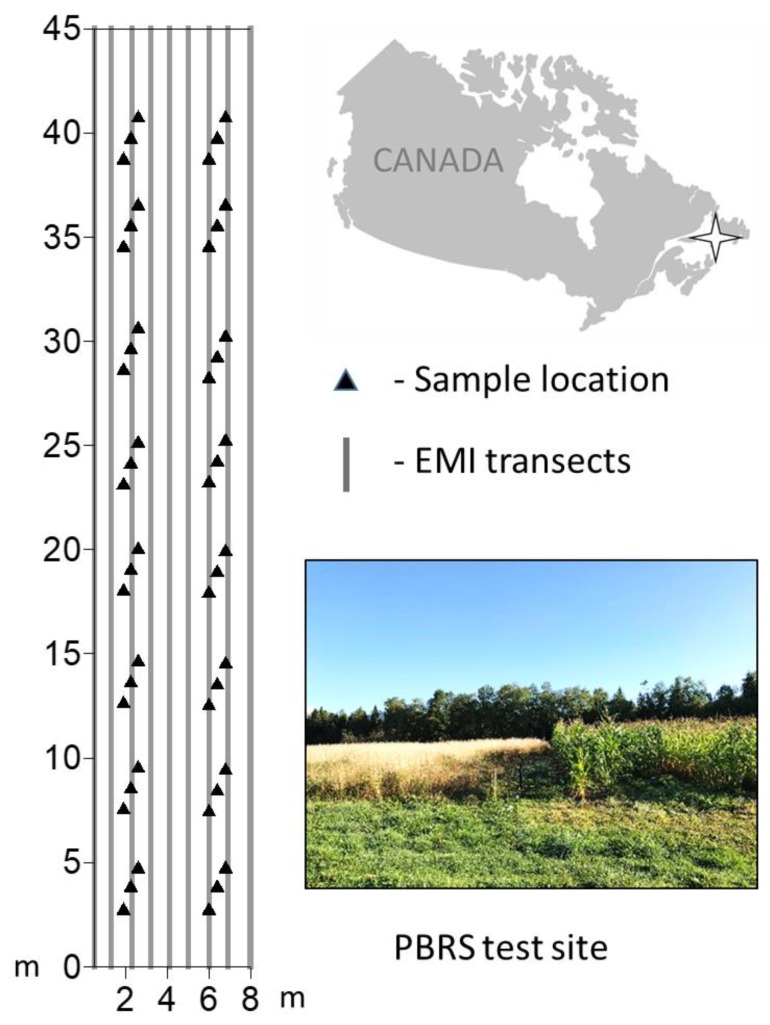
Field layout with sampling locations in the selected silage-corn field (left), location of the Pynn’s Brook Research Station (PBRS) and view on the experimental silage-corn plots during dry conditions.

The study was conducted at the Pynn’s Brook Research Station (PBRS) (49°04′23″ N, 57°33′39″ W), located in the Humber Valley, Western Newfoundland, Canada (Figure 1). The soil texture in the top 0–15 cm soil layer is sandy loam to loamy fine sand, overlain over sandy fluvial and glacio-fluvial deposits [25]. The experimental area covers approximately 0.4 ha of five different silage-corn varieties with different agronomic treatments and an adjacent grassed field [2,26]. A detailed study using EMI instruments was focused on one variety of the silage-corn experiment only, which covered approximately 350 m^2^ area. The crop was fully grown and considerably similar for both wet and dry days where EMI data collection was carried out. The mean annual precipitation and temperature obtained from the nearby weather station in Deer Lake, are 1113 mm and 4 °C, respectively, (http://climate.weather.gc.ca/). EMI and soil samples were collected on 18 August 2017 after several consecutive hot and dry days, and on 13 October 2017, after 57 days with ~174 mm of total rainfall, to represent dry and wet conditions, respectively. 

### 2.2. Soil Sampling and Analysis

We collected undisturbed and composite soil samples from the selected study plot. The undisturbed soil samples were taken from 0 to 15 cm depth and used for texture (*n* = 24) and BD (*n* = 48) analysis. Composite soil samples were collected from 0 to 20 cm to investigate the depth averaged values of SWC, CEC, pH, and EC_w_. Each composite sample comprised three samples collected in each treatment plot on a diagonal line with 1 m distance and 0.3 m spacing (Figure 1). All soil samples were analyzed according to the standard protocols (Table 1). For simplification, we assumed a homogenous distribution of the soil texture and BD within the depth of 0–20 cm with no temporal changes throughout the study period.

### 2.3. Electromagnetic Induction Surveys

#### 2.3.1. Field Data Collection

The working principle of EMI is based on a two-coil system (transmitter and receiver coil) and has been established for several decades [33,34]. A transmitter coil generates the primary magnetic field which noninvasively induces eddy currents in the soil that in turn generate a secondary magnetic field. From the ratio between both magnetic fields, the bulk EC_a_ (as integral over a certain soil volume) can be derived under low induction number conditions [33]. Meanwhile MC as well as MF sensors are commercially available, allowing for simultaneous recording of EC_a_ from different depth integrals. Two established EMI instruments were used in this study, both build to investigate the shallow soil properties: the CMD–MINIEXPLORER (GF-Instruments, Brno, Czech Republic) operating with a fixed frequency of 30 kHz and three coil separations (0.32 m, 0.71 m and 1.18 m), [11,35], and the GEM–2 (Geophex Ltd., Raleigh, NC, USA) with up to six manually set frequencies and one coil separation (1.67 m plus bucking coil at 1 m) [36,37]. EC_a_ data were recorded in vertical coplanar (VCP) and horizontal coplanar (HCP) coil orientations in both instruments. Instruments were warmed up for >20 min before data recording and held approximately 0.20 m (MC) and 1.0 m (MF) above ground according to the manufacturer instructions. We used a track distance of 1.0 m and instrument orientation parallel to the transects (Figure 1). No GPS was used. Temperature corrections for the EC_a_ were done using the temperatures values from soil probes [38].

#### 2.3.2. Theoretical Investigation Depth 

Although the investigation depths (depth of EC_a_ origin) for MC instruments are widely accepted, the related depth function is based on a theoretical equation, derived for ideal homogeneous material [34]. According to McNeill’s approximation [34], the investigation depth is related to the coil separation and its orientation to the surface. The VCP orientation has its highest sensitivity closer to the surface while the HCP orientation reaches deeper depths. The employed CMD–MINIEXPLORER provides six integral depths if both coil orientations are used. If 75% of the cumulative signal were considered, as suggested [34], this would provide EC_a_ from the following investigation depth: VCP‒C1 (25 cm), VCP‒C2 (50s cm-shallow), VCP‒C3 (90 cm), HCP‒C1 (50d cm-deep), HCP‒C2 (105 cm), and HCP‒C3 (180 cm) [11,39]; while the signal origin from the VCP is generally closer to the surface. Field experiments as well as the local sensitivity derived from the theoretical function, however, suggested that the majority of the signal response originates from a shallower depth (<100 cm for all separations and configurations) [35]. Still, given the integral characteristics of EC_a_ and the heterogeneities from natural soils, the actual signal origin in field could be varied in relation to the affecting conditions.

**Figure 2:**
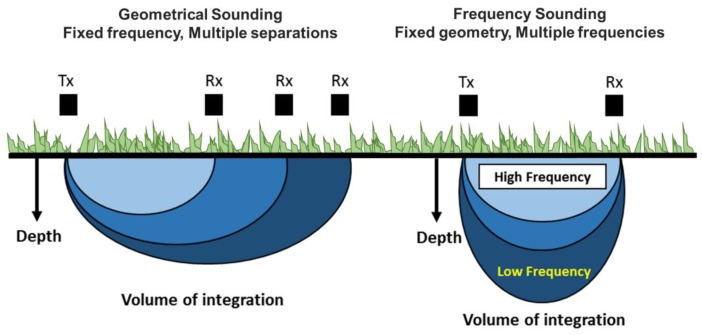
Depth sensitivity using geometry (**left**) and frequency (**right**) sounding methods of electromagnetic induction (EMI) (modified from Keiswetter and Won [41].

The depth of investigation for the MF instruments is theoretically controlled through the employed frequencies, with lower frequencies reaching greater depths (http://geophex.com). Hence, the MF principle and its possibility of changing frequencies was proposed for depth sounding [40]. The GEM–2 employed in this study allows for the simultaneous recording of up to ten frequencies. However, selection of too many frequencies reduces the strength of each frequency signal and consequently lowering the resolution. Based on previous studies, we selected three frequencies, which were also suggested by the manufactures (default settings): 18 kHz, 38 kHz, and 49 kHz. Using again both coil configurations, the GEM‒2 provided six sampling depths; hereafter these depths are denoted as VCP‒18 kHz, VCP‒38 kHz, VCP‒49 kHz, HCP‒18 kHz, HCP‒38 kHz, and HCP‒49 kHz (Figure 2).

### 2.4. EMI Data Processing

All EC_a_ were initially quality checked based on their signal-noise ratio. Thereby we considered data to be very noisy if the noise level reached a higher magnitude than the informal content in the variogram analysis. The readings from VCP‒C1 and HCP‒C1 (MC) as well as VCP‒18 kHz and HCP‒18 kHz (MF) did not pass the quality check and were consequently ignored from further processing as previously suggested [2,6,42]. EC_a_ data were interpolated by ordinary block kriging using Surfer11 software (Golden Software Inc., Golden, CO, USA) [18]. Point values for the soil sampling locations were derived from interpolated maps and averaged for each treatment plot resulting in 16 points for both dry day and wet day. Simple Pearson’s correlation (r) was calculated between soil properties and discrete EC_a_ data for each frequency, coil configuration, and coil separation, using the statistical software Minitab 17 (Minitab Inc., State College, PA, USA).

To assess the practical purpose of the mapping quality from both sensors, we used the provided correlations and predicted the targeted soil properties based on the linear models from each EC_a_ data set. We validated the model accuracies by using leave-one-out validation [11,43]. By comparing the independent prediction vs. the measured values, we displayed the coefficient of determination (R^2^) as indication for the validated explanatory power of each linear model.

Two assumptions were made in this study: (i) The quadrature component of the secondary field was proportional to EC_a_ under low induction number condition [3,39]; and (ii) soil texture and BD data were assumed to be stable over the monitoring duration and the same values were used for both dry and wet day analyses [10,44].

## 3. Results and Discussion

### 3.1. Descriptive Analysis of Soil Properties

The soil was a loamy sand with high sand content (73.2%), relatively uniformly distributed among the samples (CV = 4.7%) (Table 2). On the other hand, the variability of silt (20.8%) and clay contents (6.0%) were greater (CV of 15.3% and 13.1%, respectively). The BD of 1.4 g/cm^3^ was at the upper end of the range considered ideal for plant growth (www.nrcs.usda.gov) with relative low variation among the samples (CV 5.1%), indicating uniform compaction across the field. Except for EC_w_, all tested temporally variable soil properties were higher on the wet day compared to the dry day, SWC decreased from 19.7% to 12.3%. We interpret the lower EC_w_ on the wet day to be due to dilution effect leading to lower ionic strength of the soil solution. Moreover, the root uptake of nutrients and leaching from the bottom of the soil column will also result in low EC_w_. The CEC was relatively stable while the strongly acidic soil (pH 5.4) became moderately acidic (pH 5.7) when wet [45].

### 3.2. Descriptive Analysis for EC_a_ Data

The readings from both EMI instruments (Table 2) show relatively low EC_a_ for their sandy soil, as also reported by several previous studies [2,46]. Both instruments recorded higher EC_a_ on the wet day, most likely as consequence of higher SWC (Table 2). The MF measured relatively higher values throughout all readings which might be related to the depth of signal origin. The MF‒EC_a_ also had higher CVs (up to 58.7% for HCP‒38 kHz) than MC‒EC_a_ (<13%). The second coil separation (C2) of the MC produced the highest mean EC_a_, of 4.0 ± 0.3 mS/m) for HCP–C2 (dry) and 6.2 ± 0.8 mS/m for VCP–C2 (wet). Interestingly, the CV was higher for MC on the wet day whereas it was lower for the MF‒EC_a_. EC_a_ measured from VCP‒49 kHz was 20.3 ± 0.7 mS/m, the highest value among both instruments and coil orientations, and produced the lowest CV (3.7%). The 38 kHz frequency data of the MF showed high CVs on both days compared to all other EC_a_ values, indicating higher variability of the recordings. On the other hand, the EC_a_ measurements by 49 kHz frequency had a relatively low CV (3.7) and higher mean EC_a_ value, ranging from 7.5 (±0.7) to 20.3 (±0.7) mS/m for both days (Table 2).

For the dry day, the VCP mode of the MF EMI gives a higher EC_a_ compared to the HCP mode. A similar pattern of high variability on dry day vs. wet day for MF instruments has been reported [4,19]. Overall, for the wet day, the 38 kHz data from MF EMI, and soil properties including silt, clay, SWC, and CEC showed similar variability. Likewise, EC_a_ data measured by 49 kHz frequency showed narrow variability for the same soil properties for the dry day. All MC EMI data showed adequate variability range with the aforementioned soil properties for both days compared to MF EMI sensor.

### 3.3. Correlation to the Targeted Soil Properties

The Pearson correlation coefficient (r) between EC_a_ and soil properties (Table 3) show various significant (*p* < 0.05) correlations with the stable properties. EC_a_ from both sensors were negatively correlated to sand, while for the MC, almost every coil separation and orientation was significant. As for MF‒EC_a_ data, correlations were poor and non-significant. The negative correlations were most likely the results of the high amount of low (electric) conductivity sand content in the soil. In general, larger sand particle sizes were associated with decrease in the EC_a_ [47,48,49].

Silt was clearly and positively correlated to all EC_a_ readings from both sensors. The MC‒EC_a_ provided again higher correlations, consistently significant, while the MF‒EC_a_ readings provided significant (except HCP‒38 kHz), yet lower correlations. The positive correlations between silt and EC_a_ were previously documented [50]. Both correlations, to sand and silt, could be due to a relatively high data range of these properties. Clay content is also generally considered to have a positive relationship to EC_a_ [9,51]. However, in contrast to the majority of previous findings, the correlations to clay in our study remained insignificant for both instruments and partly even negative. One reason therefore could be found in the overall low clay content (6%) and its narrow data range, hindering positive correlations, as reported by Bronson et al. [52]. EC_a_ for both sensors had further negative and insignificant correlations to BD. Although BD is commonly considered as positively correlated to EC_a_ as a result of higher current flow because of greater particle contacts [53,54,55], it was also observed to be negative in soils with higher organic matter content [11]. Additionally, the correlation between EC_a_ and BD is related to mineral content, soil solution, and air phases resulting the interplay between the non-conductive dry mineral part and the liquid conductive phase, while only the liquid phase conducts electrical current through the soil [56]. Therefore, high BD values do not necessarily result in higher EC_a_. We explained the observed negative correlations of EC_a_ to BD at our test site by the limited amount of conductive (wet) clay minerals and the high non-conductive mineral (sand) parts, acting rather as insulation. The relative homogeneous BD among the tested site (Table 2) and its corresponding narrow data range additionally hindered to establish proper relationships with EC_a_.

The potential correlation of SWC and EC_a_ are probably the most prominent relationship and build the baseline for various SWC documented mapping studies [56]. During the dry day, SWC correlations with EC_a_ readings were positive and significantly consistent for both sensors (Table 3). The 38 kHz data of the MF even reached the highest *r* (0.83). These correlations declined for the wet day, particularly visible for the 38 kHz values recorded by the MF which was opposite to our expectations. This phenomenon could result a lower EC_w_ value in the solute phase as measured during wet conditions. The simultaneous higher correlations to sand and silt on the wet day pointed to an inflecting influence of higher SWC on the MF‒EC_a_. Higher negative correlations to sand might be caused by its insulating effect on the overall higher bulk electrical conductivity, while the higher correlations to silt were probably caused by activating of the conductive surface layers under wet conditions. In contrast, the MC‒EC_a_ values were relatively stable for both days, providing moderate to strong and always significant correlations to SWC.

While pH could not be related to any of the EC_a_ sets, CEC on the dry day had significant influence for both instruments. However, CEC significantly correlated only VCP‒C3 and HCP‒C2 of MC data on the wet day, while other MC and all the MF recordings remained insignificant. We overall explained the weaker correlations on the wet day with lower CEC values and spatial variabilities (Table 2) limiting its allocation to the EC_a_. On the dry day, the correlation to EC_w_ were positive and partly significant to the MC values only, while its influence on the MF was negligible. Under the wet condition, all relationships of EC_a_ to EC_w_ were higher for both sensors, but significant only to VCP‒49 kHz (MF), VCP‒C3, and HCP‒C2.

We explained the higher correlation from EC_a_ to EC_w_ during wet conditions by the accompanying higher SWC resulting in a higher saturation percentage and potential dissolution of ions to the soil solution potentially increasing the ionic strength. Since the soil had not reached the saturation of 47.2% on the wet day, based on hydrological simulations [57], there was no chance for ions to be leached out from the soil lowering the EC_w_. In contrast to the hypothesis and the previous findings [6], the correlations to SWC under wet conditions were lower or similar. This phenomena of higher correlation between EC_a_ and SWC on drier days was also observed in sandy soils [58]. Although higher EC_w_ values were empirically related to deflecting the EC_a_–SWC correlations [17,53], which was also observed under field conditions [6], very low EC_w_ values demonstrated as narrowing the EC_a_–SWC relationship, which could lead to lower prediction accuracy [56]. The higher EC_a_–SWC correlations on the dry day might be the result of the higher EC_w_. On the other hand, the correlation of EC_a_ to EC_w_ were higher for the wet day, despite the narrow EC_w_ data range, indicating an interplaying effect of SWC and its ionization on the recorded EC_a_ [56]. Regardless of the differences between the sensors, the analysis highlights the complexity of signal response and its interactions between, free water, absorbed water, EC_w_, and particles content [56]. However, because of the limited data in this study, we are not capable to explain the mixed behavior of EC_w_ under different sensors and coil orientation in total, which needs to be investigated in future studies.

Considering the explanatory power of the EC_a_ correlations in Table 3 and its practical use for field mapping applications, Table 4 shows the accuracies from the respective linear models by means of the R^2^. The R^2^ was generated by the predicted vs. the measured soil properties using the leave-one-out validation. Although the r in Table 3 reveled several promising significant correlations, their practical applications under field conditions remain limited. Using these correlations, only the SWC model based on VCP‒38 kHz and HCP‒C3 on the dry day and VCP‒C3 on the wet day, as well as CEC from HCP‒C2 and HCP‒C3 on the wet day reached prediction accuracies higher up to 50%. Clay, BD, and pH accuracies were zero or negligible for both sensors. Prediction accuracies for sand ranged between 4.4% (VCP‒49 kHz–dry day) and 13.8% (VCP‒49 kHz-wet day) for the MF and 27.5% to 38% (HCP‒C2, VCP‒C2–dry day) and 17.1% to 36.3% (HCP‒C2, VCP‒C3-wet day) for the MC. Similar higher predictions of the MC-based models were observed for silt in both days, reaching up to 29% for the MF (VCP‒49 kHz-wet day) while up to 45.3% for the MC (VCP‒C3-wet day). However, we like to emphasize that the limited amount of samples restricted the overall prediction quality of all models. To archive sufficient accuracies for practical predictions, higher samples amounts would be required which was however not the focus of this study.

With respect to the overall results, the MC sensor performed better for the tested podzolic soil and selected variables, providing more stable EC_a_ data sets and higher correlations to the targeted soil properties. With the exception of one single data set (SWC vs. VCP‒38 kHz on the dry day), all correlations were lower and less significant for the MF sensor. The results further suggested a higher susceptibility of the MF–EC_a_ to SWC variation, which could limit its operation for SWC mapping. However, we need to emphasize that our study considered variables from one soil type and shallow depths only.

## 4. Conclusions

We performed a comparative study using MF and MC EMI sensors to assess their ability for mapping properties for shallow soils under dry and wet conditions as relevant for agricultural purposes. The results show significant (*p* < 0.05) positive correlations to silt, SWC and CEC to EC_a_ from both sensors under dry conditions, with higher correlations and significant levels for the MC sensor. The correlations of the MF–EC_a_ to temporal variable properties SWC and CEC became insignificant under wet conditions, except for one frequency (38 kHz) to SWC. However, wet conditions increased the correlation of MF–EC_a_ to sand and silt opposite to our expectations. In contrast, the correlations of the MC–EC_a_ sensor to sand, silt, SWC and CEC remained relatively stable under both, dry and wet conditions. Sand was negatively correlated for both sensors on the dry day, however, only significant for the MC reading for both days. No significant correlations to clay, BD, and pH were found for either instrument, likely because of their limited amount and variability in the tested soil. Likewise, the prediction accuracies based on the linear correlations were lower for the MF sensor. From all considered variables and correlations, only SWC and CEC were reasonably projected (R^2^ > 0.5) if EC_a_-based models were used for independent prediction of its variation, contrary to our hypothesis. 

Given the results presented here, we concluded that the MF sensor is more affected by temporally variable soil properties, in particular by variation in SWC, which influenced its ability to establish proper relationships to these targeted variables (except 38 kHz) but also affecting its ability to map stable properties. The performance of the MC sensor was less affected by different weather conditions, providing overall stronger correlations to both, stable and temporally variable soil properties. At the tested loamy sand, the EC_a_ data from the MC sensor were more suitable to investigate the spatiotemporal variability of shallow, agriculturally relevant soil properties compared to the MF sensor. Similar tests for different soil types and management conditions are needed to further verify these findings.

## Figures and Tables

**Table 1:** Soil property measured, instrument and the method used.

Soil Property	Instrument	Standard Method
Soil texture	Standard hydrometer (ASTM, USA)	Hydrometer method [27]
BD (g/cm^3^)	Core sampler with a sliding hammer	Core method [28]
SWC (%)	Convection Oven (Thermo Scientific, USA)	Gravimetric with oven drying [29]
CEC (cmol/kg)	Ion Chromatography- Dionex^TM^ ICS-5000^+^ DC-5 Detector/Chromatography (Thermo Scientific, Waltham, MA,USA)	Sodium Acetate method-EPA 9081 [30]
pH	HI9813-6 portable pH/EC/TDS/Temperature meter (HANNA instruments, Woonsocket, RI, USA)	0.01 M CaCl_2_ method [31]
EC_w_ (mS/cm)	HI9813-6 portable pH/EC/TDS/Temperature meter (HANNA instruments, Woonsocket, RI, USA)	EC_1:2,_ soil: deionized water [32]

ASTM−American Society for Testing and Materials; EPA−Environmental Protection Agency; EC−electrical conductivity; TDS−total dissolved solids; M−molarity of the solution.

**Table 2:** Descriptive statistics of soil properties and EMI‒EC_a_ (mS/m) data for both dry and wet days (*n* = 16).

	Dry Day					Wet Day				
Variable	Mean	SD	CV	Min	Max	Mean	SD	CV	Min	Max
**Soil properties**										
**Sand (%)**	74.2	3.5	4.7	68.0	81.7	-	-	-	-	-
**Silt (%)**	19.8	3.1	15.3	13.7	25.4	-	-	-	-	-
**Clay (%)**	6.0	0.8	13.1	4.7	7.5	-	-	-	-	-
**BD (g/cm^3^)**	1.4	0.1	5.1	1.3	1.5	-	-	-	-	-
**SWC (%)**	12.3	1.6	12.9	9.3	15.5	19.7	3.0	15.0	15.1	23.8
**pH**	5.4	0.2	3.7	4.9	5.7	5.7	0.2	4.2	5.3	6.1
**CEC (cmol/kg)**	11.0	2.1	19.3	8.0	14.3	12.2	1.9	15.8	9.4	15.1
**EC_w_ (mS/m)**	20	10	41.2	10	50	10	0.0	26.8	10	10
**MF**‒**EMI**										
**VCP‒38 kHz**	1.9	0.8	39.2	0.9	3.3	3.9	0.7	18.5	2.8	5.2
**VCP‒49 kHz**	11.4	1.1	9.2	9.5	13.5	20.3	0.7	3.7	19.1	21.8
**HCP‒38 kHz**	1.6	1.0	58.7	0.7	3.8	6.3	0.8	12.8	5.2	7.7
**HCP‒49 kHz**	7.5	0.7	9.5	6.6	8.8	16.6	0.7	4.2	15.7	17.9
**MC**‒**EMI**										
**VCP‒C2**	3.4	0.3	7.5	2.9	3.9	6.2	0.8	12.8	5.3	7.7
**VCP‒C3**	3.1	0.3	8.0	2.6	3.5	3.5	0.4	11.0	2.7	4.1
**HCP‒C2**	4.0	0.3	6.6	3.6	4.5	4.4	0.4	9.0	3.7	5.0
**HCP‒C3**	3.6	0.3	8.9	3.1	4.1	4.2	0.4	10.2	3.5	5.1

SD‒standard deviation; CV‒coefficient of variation (%); Min‒minimum; Max–maximum, all values were rounded for one decimal.

**Table 3:** Pearson’s correlation coefficient (r) summary between soil properties (0–20 cm depth), and temperature corrected EC_a_ data for both wet and dry days (*n* = 16), abbreviations are explained in Section 2.3.1 under field data collection.

	VCP‒38 kHz	VCP‒49 kHz	HCP‒38 kHz	HCP‒49 kHz	VCP‒C2	VCP‒C3	HCP‒C2	HCP‒C3
**Dry day**								
Sand (%)	−0.48	−0.48	−0.34	−0.41	−0.75 ***	−0.69 **	−0.68 **	−0.43
Silt (%)	0.61 *	0.59 *	0.48	0.55 *	0.73 ***	0.72 **	0.73 ***	0.55 *
Clay (%)	−0.26	−0.20	−0.38	−0.33	0.45	0.20	0.18	−0.24
BD (g/cm^3^)	−0.40	−0.150	−0.17	−0.40	−0.16	−0.33	−0.34	−0.46
SWC (%)	0.83 ***	0.50 *	0.65 **	0.76 ***	0.55 *	0.74 ***	0.71 **	0.79 ***
pH	−0.17	−0.33	−0.06	−0.16	0.10	0.02	−0.22	−0.20
CEC (cmol/kg)	0.70 **	0.51 *	0.61 *	0.65 **	0.60 *	0.77 **	0.79 ***	0.78 ***
EC_w_ (mS/cm)	0.21	0.005	0.11	0.062	0.47	0.44	0.60 *	0.38
**Wet day**								
Sand (%)	−0.38	−0.60 *	−0.41	−0.47	−0.48	−0.72 **	−0.61 *	−0.53 *
Silt (%)	0.51 *	0.69 **	0.55 *	0.60 *	0.62 **	0.76 ***	0.66 **	0.62 **
Clay (%)	−0.31	−0.07	−0.35	−0.29	−0.29	0.24	0.11	−0.06
BD (g/cm^3^)	−0.43	−0.28	−0.33	−0.37	−0.37	−0.28	−0.34	−0.39
SWC (%)	0.47	0.63 **	0.47	0.56 *	0.55 *	0.81 ***	0.77 ***	0.68 **
pH	0.09	−0.08	−0.03	−0.10	−0.07	−0.15	−0.11	0.02
CEC (cmol/kg)	0.25	0.43	0.29	0.39	0.37	0.68 **	0.63 **	0.49
EC_w_ (mS/cm)	0.37	0.60 *	0.39	0.37	0.38	0.63 **	0.50 *	0.46

Bold numbers correspond to significant correlations (*** *p* < 0.001, ** *p* < 0.01, * *p* < 0.05) BD‒bulk density; SWC‒soil water content (gravimetric); CEC‒cation exchange capacity; EC_w_‒pore water electrical conductivity

**Table 4:** Coefficient of determination (R^2^) of the leave-one-out validation as obtained by the linear models between the soil properties (0–20 cm depth), and temperature corrected EC_a_ data for both wet and dry days as displayed in Table 3 (*n* = 16).

	VCP–38 kHz	VCP–49 kHz	HCP–38 kHz	HCP–49 kHz	VCP–C2	VCP–C3	HCP–C2	HCP–C3
**Dry day**								
Sand (%)	0	0.044	0	0	0.38	0.293	0.275	0
Silt (%)	0.153	0.195	0	0.109	0.346	0.378	0.354	0.122
Clay (%)	0	0	0	0	0	0	0	0
BD (g/cm^3^)	0	0	0	0	0	0	0	0
SWC (%)	**0.571**	0.072	0.223	0.411	0.07	0.384	0.296	0.506
pH	0	0	0	0	0	0	0	0
CEC (cmol/kg)	0.384	0.1	0.221	0.266	0.203	0.471	**0.507**	**0.518**
EC_w_ (mS/cm)	0	0	0	0	0	0	0.084	0
**Wet day**								
Sand (%)	0	0.138	0	0	0	0.363	0.171	0
Silt (%)	0.075	0.29	0.105	0.147	0.166	0.453	0.278	0.138
Clay (%)	0	0	0	0	0	0	0	0
BD (g/cm^3^)	0	0	0	0	0	0	0	0
SWC (%)	0	0.175	0.001	0.096	0.078	**0.567**	0.473	0.204
pH	0	0	0	0	0	0	0	0
CEC (cmol/kg)	0	0	0	0	0	0.327	0.228	0
EC_w_ (mS/cm)	0	0.08	0	0	0	0.192	0	0

Bold numbers correspond to correlations R^2^ > 0.5
